# Prevalence, risk factors, and outcomes of malnutrition in older adults hospitalized with community-acquired pneumonia: a retrospective study

**DOI:** 10.3389/fmed.2025.1522261

**Published:** 2025-04-30

**Authors:** Xiao-lu Zheng, Xing Zhang, Jia-hui Yuan, Lu-tian Yi

**Affiliations:** ^1^Department of Respiratory and Critical Care Medicine, The Affiliated People’s Hospital of Ningbo University, Ningbo, Zhejiang, China; ^2^Department of Thyroid and Breast Surgery, The Affiliated People’s Hospital of Ningbo University, Ningbo, Zhejiang, China

**Keywords:** risk factors, malnutrition, community-acquired pneumonia, older adults, prevalence

## Abstract

**Purpose:**

Community-acquired pneumonia (CAP) is a common health problem in older adults. Malnutrition is also prevalent in the elderly population. This study aimed to investigate the prevalence, risk factors, and outcomes of malnutrition in hospitalized older adults diagnosed with CAP.

**Methods:**

From April 2023 to October 2023, clinical data of older adults hospitalized with CAP were retrospectively analyzed. Based on their malnutrition status at the time of admission, patients were classified into the malnutrition and non-malnutrition groups. The demographic and clinical characteristics as well as outcomes were compared between the two groups. Univariate and multivariate logistic regression analyses were used for variables of interest. The receiver operating characteristic curve was applied to evaluate the risk factors.

**Results:**

A total of 511 eligible patients were included in this study. There were 90 and 421 patients in the malnutrition and non-malnutrition groups, respectively. Univariate analysis showed a significant difference in six factors: age, living alone, past cerebral stroke, Parkinson’s disease, serum albumin, and hemoglobin (*P* < 0.05). Multivariate analysis revealed that age (OR = 1.044, *P* = 0.025), past cerebral stroke (OR = 2.643, *P* = 0.014), Parkinson’s disease (OR = 2.998, *P* = 0.028), low serum albumin level (OR = 6.407, *P* < 0.001), and low hemoglobin level (OR = 4.629, *P* < 0.001) were independent risk factors for malnutrition. Receiver operating characteristic curve analysis of age, serum albumin level, and hemoglobin level showed a cutoff value of 74 years, 40.5 g/L, and 105 g/L, respectively.

**Conclusion:**

The prevalence of malnutrition was high in older adults hospitalized with CAP. Malnutrition was associated with worse outcomes, including prolonged hospital stay, higher in-hospital mortality, and increased readmission. Old age (> 74 years), past cerebral stroke, Parkinson’s disease, low serum albumin level (< 40.5 g/L), and low hemoglobin level (< 105 g/L) were regarded as independent risk factors.

## 1 Introduction

Community-acquired pneumonia (CAP) is a common health problem with high morbidity and mortality rates in older patients. In China, despite a significant reduction in deaths due to CAP between 1990 and 2013, it remains a significant threat to older adults, is a leading cause of death, and is responsible for more than 19 deaths per 100,000 people annually (1, 2). In the USA, after 70 years of commercial use of antibiotics, the ranking of pneumonia as a leading cause of death has only dropped from 6th in 1946 to 8th in 2016 (3, 4). Despite medical advances, CAP remains one of the leading causes of hospitalization for infections in older patients and is associated with high medical costs worldwide (5). Concurrently, aging is associated with immunosenescence, chronic comorbidities, and structural lung changes, increasing susceptibility to severe infections (6).

Malnutrition is reportedly highly prevalent in the older population (7). Nutrition is a determinant of health but is easily overlooked in infectious diseases, (8) and patients are susceptible to disease progression. Several studies have focused on chronic disease-related malnutrition and have shown that malnutrition affects 30–50% of hospitalized elderly patients (9–11). Malnutrition exacerbates immune dysfunction by impairing lymphocyte proliferation, phagocytic activity, and antibody production, while also causing muscle wasting—including respiratory muscles critical for airway clearance (12). Malnutrition has been associated with negative effects on several diseases, including the risk of infection and complications, prolonged hospital stay, increased morbidity and mortality, and impaired functional recovery (13–15). However, malnutrition in older patients with CAP has not been sufficiently studied.

This study aimed to investigate the prevalence, risk factors, and outcomes of malnutrition in hospitalized older patients with CAP. We hope to provide a basis for the early clinical detection and avoidance of malnutrition.

## 2 Materials and methods

### 2.1 Study design and eligibility

This retrospective observational study was conducted at the Affiliated People’s Hospital of Ningbo University, a large tertiary hospital in Zhejiang Province, located on the densely populated eastern coast of China. Medical records of hospitalized patients diagnosed with pneumonia between April and October 2023 were reviewed. The inclusion criteria were as follows: (1) age > 65 years; (2) diagnosis of CAP; and (3) complete nutritional assessment records. The exclusion criteria were: (1) acute infections in other organs; (2) acute cardiovascular disease; (3) acute cerebrovascular disease; (4) acute liver failure; (5) acute kidney injury; (6) active malignant tumors; (7) incomplete medical records; (8) lack of routine laboratory variables; (9) lack of computed tomography (CT) scan of the chest. CAP was diagnosed in accordance with the Chinese Guidelines for Adult CAP (2016 edition) (16). This study was conducted in accordance with the requirements of the Declaration of Helsinki of the World Medical Association and was approved by the Ethics Committee of the Affiliated People’s Hospital of Ningbo University (2024-091).

### 2.2 Nutritional assessment method

The Mini Nutritional Assessment Tool (MNA) was used to identify malnutrition and the risk of malnutrition as recommended by the European Society of Parenteral and Enteral Nutrition for nutritional assessment in older patients (17). The MNA is a simple, non-invasive, and effective malnutrition screening tool. It contains 18 items covering four dimensions (18). Briefly, the screening and assessment of the MNA include the following: anthropometry containing body mass index, mid-arm circumference, calf circumference, and weight loss during the last 3 months; dietary assessment (daily number of meals, protein intake, fruit and vegetable intake, appetite, fluid intake, and mode of feeding); general assessment (lives independently, mobility, prescribed medication, neuropsychological disorders, psychological stress or acute disease in the past 3 months, and pressure sores or skin ulcers), and a subjective assessment (health status compared with other people of the same age and food intake decline). The MNA has a total score of 30 points, which was divided into two categories in this study: malnutritional status (score < 24) and non-malnutritional status (score ≥ 24). Accordingly, patients diagnosed with CAP were classified into malnourished and non-malnourished groups.

### 2.3 Treatment regimen

Patients treated for CAP received antimicrobials with empirical coverage normally including a β-lactam plus a macrolide or a respiratory fluoroquinolone alone within 6 h of their arrival to the hospital. Antimicrobial treatment was adjusted according to identification and sensitivity information or clinical indicators. The initial treatment was administered intravenously to inpatients and switched to oral antimicrobial therapy after the patients became stable.

### 2.4 Discharge criteria

Patients with CAP were discharged if the following criteria were met: body temperature < 37.2°C for at least 3 days; clinical symptoms such as fever, cough, sputum, and wheezing had significantly improved or even disappeared; negative blood or sputum culture; white blood cell count < 12*10^9^/L on routine blood tests; chest CT examination revealed significant or complete absorption of the inflammatory lesions in the lungs compared to admission; respiratory rate < 24 breaths/min; blood oxygen saturation more than 90% in room air; and the patients ate and drank adequately on their own in the past 24 h.

### 2.5 Data collection

All patients were evaluated using the MNA, and scores were calculated on admission. The following data were collected: age, sex, living alone status, smoking status, daily alcohol intake, duration of symptoms before admission, medical history, and comorbidities. Pretreatment laboratory indicators and radiological findings were collected from medical records, including serum albumin, hemoglobin, serum sodium, serum potassium, C-reactive protein, infectious pathogens, multidrug-resistant bacterial infection, and involvement of the lung lobes. The treatment outcomes, including the duration of antibiotic treatment, hospital stay, in-hospital death, and readmission within 30 days, were also documented.

### 2.6 Statistical analysis

Continuous variables were expressed as mean ± standard deviation and compared using independent *t*-tests or the Mann-Whitney U test between the two groups. For categorical data, the chi-square test or Fisher’s exact test was used. Univariate exact logistic regression analysis was performed to identify the risk factors for malnutrition. Significant variables (*P* < 0.10) were identified in the multivariate logistic regression analyses. The receiver operating characteristic (ROC) curve was used to evaluate the risk factors of the binary logistic model, and cutoff values for the corresponding risk factors were obtained. All statistical analyses were performed using SPSS version 22.0 (IBM Corp., Armonk, NY, USA), with statistical significance set at *P* < 0.05.

## 3 Results

### 3.1 Baseline characteristics

**FIGURE 1:**
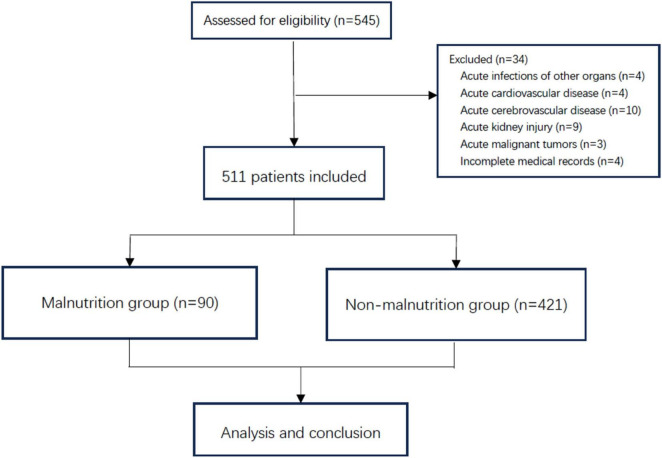
Flowchart of patient enrollment.

Overall, 511 patients were included in this study, with an average age of 71.8 ± 6.7 years, and 90 and 421 patients were included in the malnutrition and non-malnutrition groups, respectively. In this cohort, 17.6% of the patients had malnutrition. The study algorithm and analysis process are shown in Figure 1.

The demographic and clinical characteristics of the patients were compared between the two groups. There were no significant differences between the two groups in terms of sex, smoking, daily alcohol intake, duration of symptoms before admission, cancer history, myocardial infarction, hypertension, diabetes, hypercholesterolemia or hyperlipidemia, Alzheimer’s disease, liver dysfunction, renal insufficiency, chronic bronchitis, chronic obstructive pulmonary disorder, or immunosuppression (*P* > 0.05). However, statistically significant differences were found in age, living alone, history of cerebral stroke, and Parkinson’s disease (*P* < 0.05) (Table 1).

**TABLE 1:** Demography and clinical characteristics of patients between the two groups.

	Malnutrition group (*n* = 90)	Non-malnutrition group (*n* = 421)	*P*-value
Age (years)	73.4 ± 9.0	71.5 ± 6.0	0.013
Sex (*n*, %)			0.287
Male	46 (51.1%)	241 (57.2%)	
Female	44 (48.9%)	180 (42.8%)	
Living alone (*n*, %)	27 (30%)	70 (16.6%)	0.003
Smoking (*n*, %)	10 (11.1%)	52 (12.4%)	0.744
Daily alcohol intake (*n*, %)	9 (10%)	50 (11.9%)	0.613
DSBA (days)	2.8 ± 1.6	2.9 ± 1.7	0.710
**Past medical history (*n*, %)**			
Cancer	12 (13.3%)	39 (9.3%)	0.242
Cerebral stroke	15 (16.7%)	28 (6.7%)	0.002
Myocardial infarction	6 (6.7%)	33 (7.8%)	0.704
**Comorbidity (*n*, %)**			
Hypertension	22 (24.4%)	107 (25.4%)	0.847
Diabetes	12 (13.3%)	44 (10.5%)	0.427
Hypercholesterolemia or hyperlipidemia	33 (36.7%)	170 (40.4%)	0.513
Alzheimer’s disease	7 (7.8%)	20 (4.8%)	0.244
Parkinson’s disease	9 (10.0%)	18 (4.3%)	0.028
Liver dysfunction	12 (13.3%)	37 (8.8%)	0.184
Renal insufficiency	9 (10%)	30 (7.1%)	0.351
Chronic bronchitis	13 (14.4%)	45 (10.7%)	0.308
COPD	15 (16.7%)	82 (19.5%)	0.537
Immunosuppression	6 (6.7%)	21 (5.0%)	0.518

COPD, chronic obstructive pulmonary disorder; DSBA, duration of symptoms before admission.

### 3.2 Clinical indicators

Laboratory indicators and radiological findings were compared between the malnutrition and non-malnutrition groups. There were no significant differences between the two groups in terms of serum sodium, potassium, and C-reactive protein levels, pathogens, multidrug-resistant bacterial infection, or lung lobe involvement (*P* > 0.05). However, the serum albumin and hemoglobin levels were statistically significant (*P* < 0.05) (Table 2).

**TABLE 2:** Laboratory indicators and radiological findings of patients between the two groups.

	Malnutrition group (*n* = 90)	Non-malnutrition group (*n* = 421)	*P*-value
Serum albumin (g/L)	36.7 ± 5.7	44.7 ± 7.7	< 0.001
Hemoglobin (g/L)	96.4 ± 15.6	111.8 ± 19.3	< 0.001
Serum sodium (mmol/L)	139.8 ± 4.1	139.4 ± 4.3	0.457
Serum potassium (mmol/L)	4.2 ± 0.5	4.5 ± 0.3	0.925
C-reactive protein (mg/L)	44.4 ± 25.6	42.1 ± 24.0	0.401
**Pathogens (*n*, %)**			
Streptococcus pneumoniae	11 (12.2%)	45 (10.7%)	0.673
Mycoplasma pneumoniae	8 (8.9%)	28 (6.7%)	0.451
Haemophilus influenzae	6 (6.7%)	26 (6.2%)	0.861
Influenza A/B	9 (10%)	46 (10.9%)	0.797
Multidrug-resistant bacterial infection (*n*, %)	5 (5.6%)	19 (4.5%)	0.592
Involvement of lung lobes (*n*, %)			0.150
Unilateral	72 (80.0%)	362 (86.0%)	
Bilateral	18 (20.0%)	59 (14.0%)	

### 3.3 Risk factors for malnutrition

**FIGURE 2:**
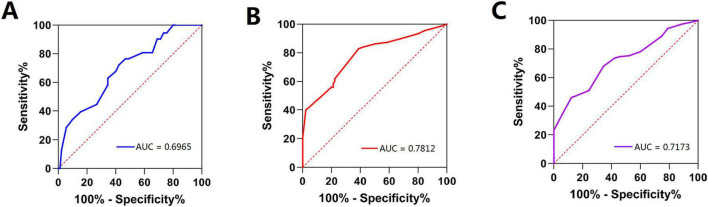
ROC curves of logistic model. ROC, receiver operating characteristic. **(A)** age; **(B)** serum albumin level; **(C)** hemoglobin level.

ROC curves were constructed for age and serum albumin and hemoglobin levels. The areas under the curves were 0.6965, 0.7812, and 0.7173, respectively (Figure 2). The cutoff values for age, serum albumin level, and hemoglobin level were set at 74 years, 40.5 g/L, and 105 g/L, respectively. Univariate analysis was conducted on variables that demonstrated statistical significance, including age (> 74 years), living alone, past cerebral stroke, Parkinson’s disease, serum albumin level (< 40.5 g/L), and hemoglobin level (< 105 g/L) (Table 3). Variables with values of *P* < 0.10 were subjected to further multivariate logistic regression analysis. We found that age (> 74 years) [odds ratio (OR) 1.044, confidence interval (CI) 1.005-1.084, *P* = 0.025], past cerebral stroke (OR 2.643, CI 1.215-5.751, *P* = 0.014), Parkinson’s disease (OR 2.998, CI 1.127-7.980, *P* = 0.028), serum albumin level (< 40.5 g/L) (OR 6.407, CI 3.611-11.366, *P* < 0.001), and hemoglobin level (< 105 g/L) (OR 4.629, CI 2.721-7.877, *P* < 0.001) were independent risk factors for malnutrition in older patients hospitalized with CAP (Table 4).

**TABLE 3:** Univariate logistic regression for risk factors for malnutrition in older adults hospitalized with community-acquired pneumonia.

	B	SE	Wals	OR	95% CI	*P*-value
Age (> 74 years)	0.046	0.018	6.379	1.047	1.010–1.084	0.012
Living alone	0.075	0.265	8.355	2.149	1.279–3.610	0.004
Past cerebral stroke	1.032	0.344	9.009	2.807	1.431–5.508	0.003
Parkinson’s disease	0.911	0.426	4.576	2.488	1.079–5.733	0.032
Serum albumin level (< 40.5 g/L)	1.732	0.273	40.353	5.652	3.312–9.645	< 0.001
Hemoglobin level (< 105 g/L)	1.394	0.245	32.340	4.032	2.494–6.520	< 0.001

**TABLE 4:** Multivariate logistic regression for risk factors for malnutrition in older adults hospitalized with community-acquired pneumonia.

	B	SE	Wals	OR	95% CI	*P*-value
Age (> 74 years)	0.043	0.019	5.015	1.044	1.005–1.084	0.025
Living alone	0.384	0.305	1.568	1.468	0.808–2.670	0.208
Past cerebral stroke	0.972	0.397	6.005	2.643	1.215–5.751	0.014
Parkinson’s disease	1.098	0.499	4.833	2.998	1.127–7.980	0.028
Serum albumin level (< 40.5 g/L)	1.857	0.293	40.315	6.407	3.611–11.366	< 0.001
Hemoglobin level (< 105 g/L)	1.532	0.271	31.927	4.629	2.721–7.877	< 0.001

### 3.4 Comparison of outcomes

Neither group exhibited significant differences in intensive care admission or life-support ventilation (*P* > 0.05). However, the malnutrition group had a longer hospital stay (9.7 ± 1.2 vs. 8.3 ± 2.1 days, *P* < 0.001), higher in-hospital mortality (6.7% vs. 1.2%, *P* = 0.001), and higher readmission rate within 30 days (10.0% vs. 4.3%, *P* = 0.028) than those in the non-malnutrition group (Table 5).

**TABLE 5:** Comparison of outcomes between the two groups.

	Malnutrition group (*n* = 90)	Non-malnutrition group (*n* = 421)	*P*-value
Intensive care (*n*, %)	5 (5.6%)	20 (4.8%)	0.748
Life support (*n*, %)	7 (7.8%)	32 (7.6%)	0.954
Hospital stay (days)	9.7 ± 1.2	8.3 ± 2.1	< 0.001
In-hospital death (*n*, %)	6 (6.7%)	5 (1.2%)	0.001
Readmission within 30 days (*n*, %)	9 (10.0%)	18 (4.3%)	0.028

## 4 Discussion

This study investigated the prevalence, risk factors, and outcomes of malnutrition in hospitalized older patients with CAP. The independent risk factors were old age (> 74 years), past cerebral stroke, Parkinson’s disease, low serum albumin levels (< 40.5 g/L), and low hemoglobin levels (< 105 g/L). Additionally, malnutrition was associated with worse outcomes, including prolonged hospital stay, higher in-hospital mortality, and increased readmission rates.

The proportion of the world’s older population continues to increase because of increased life expectancy and progress in healthcare (19). Adequate nutrition is a prerequisite for healthy aging. However, geriatric patients are predisposed to the risk of malnutrition. Malnutrition leads to various adverse consequences, such as weakness, decreased immunity, increased risk of chronic diseases, and death (20). The nutritional status of older adults is affected by various physiological, psychological, economic, and social factors. Unfortunately, although the prevalence of malnutrition is high among older adults, there is a lack of awareness in clinical practice.

CAP is the most common infection in older adults, with approximately 2 million older patients developing CAP, and it causes 1.5 million hospitalizations in the USA annually (21, 22). In China, CAP also has a high incidence, and the burden of this disease is growing substantially as the population ages (23). CAP is a leading cause of mortality and incurs significant healthcare costs. Therefore, malnutrition in older patients hospitalized for CAP should be investigated.

Nutritional assessment and intervention should be initiated in older patients with CAP admitted to hospitals. There are several nutritional screening tools, such as the Short Nutritional Assessment Questionnaire, Malnutrition Universal Screening Tool, Malnutrition Screening Tool, MNA, and Patient-Generated Subjective Global Assessment (24). We used the MNA score because it has been widely adopted for screening and assessing malnutrition in clinical practice, particularly in older patients (25). The MNA score can effectively assess nutritional status in older patients to facilitate timely and accurate detection of malnutrition and is a well-defined and easy-to-use nutritional assessment method with a sensitivity of 96% and a specificity of 98% (26). It is a nutritional assessment method designed based on the characteristics of older adults and is used as the gold standard for evaluating malnutrition in older adults. The evaluation process can be completed in several minutes, and it is easy to obtain the information from patients.

The prevalence of malnutrition is higher in hospitals than in the general community. In this study, the overall prevalence of malnutrition risk (17.6% with MNA score < 24) was lower than the reported prevalence of 28%-39.4% in the literature (27, 28). This may be because the patients included in this cohort lived in Ningbo City, located in the eastern coast of China, with good socioeconomic status. Different economic levels may contribute to different nutritional statuses (29). In this prosperous city, there is abundant availability of nutritious food, and most people know to eat a balanced diet or are aware of the “diet pagoda for Chinese residents,” thanks to health promotions advocated by the government.

In this study, we found that advanced age (> 74 years) was independently associated with poor nutritional status in older patients with CAP. The nutritional status of older patients declines with age, and the prevalence of malnutrition increases with age (30, 31). This decline in nutritional status may be associated with various factors, such as mobility impairment, tooth loss, nutrient malabsorption, impaired swallowing function, loss of appetite, and dysfunctional olfactory and taste (20, 32, 33). In the older population, routine nutritional screening can rapidly identify malnutrition, and further nutritional assessments need to be applied quickly to evaluate nutritional and metabolic status and assist in nutritional care plans.

Previous cerebral stroke and Parkinson’s disease were confirmed as independent risk factors for malnutrition in patients with CAP. The underlying causes are as follows: > 50% of stroke survivors and > 80% of patients with Parkinson’s disease develop chronic dysphagia during the course of their disease (34–36). Swallowing involves multiple muscles and nerves that depend on the central nervous system for sensory feedback, motor programming and execution, and cognitive cortical processing (37). Swallowing impairment reduces quality of life and intake of energy, water, and other nutrients, and has been identified as a risk factor for malnutrition in previous studies (38, 39). Additionally, more than 20% of patients with Parkinson’s disease are unaware of their swallowing disorders (40).

In this study, low serum albumin (< 40.5 g/L) and hemoglobin levels (< 105 g/L) on admission were significantly associated with malnutrition in patients with CAP. Low serum albumin levels during acute infection are correlated with the underlying inflammatory process and can indicate disease severity. Therefore, albumin is generally considered a negative acute-phase protein (41). The liver synthesizes serum albumin and it is often used as a marker to assess nutritional status, particularly protein-energy malnutrition. Admission serum albumin level is a strong prognostic indicator of a 40% increase in mortality in patients with low albumin concentrations and should be viewed as a marker for nutritional interventions (42). Low hemoglobin levels as a risk factor for malnutrition in patients aligns with findings from other studies (8, 25). Malnutrition can significantly affect hemoglobin levels in various ways. Deficiency in iron, protein, vitamins, or calories is a common cause of decreased hemoglobin production.

Malnutrition in older adults is a significant risk factor for poor clinical outcomes, causing a massive burden on medical resources and society (7). Herein, we found that malnutrition was associated with poor outcomes in older patients with CAP, including prolonged hospital stay, increased in-hospital mortality, and increased readmission within 30 days. These findings strongly support the need for physicians to identify malnutrition in daily clinical practice. Early screening of older patients hospitalized with CAP for malnutrition may help identify those at risk of adverse clinical outcomes who may benefit from prompt nutritional interventions. Therefore, the overall prognosis of this population may have improved.

This study had several limitations. First, this was a retrospective cohort study conducted at a single center. Second, the sample size was limited, and selection bias in the variables was inevitable. Third, the use of the MNA alone to assess the nutritional status of patients may not be objective. Finally, the evidence for the benefits of early nutritional support in older patients with CAP remains unclear. Therefore, future prospective studies with larger sample sizes, more variables, and combined nutritional assessment tools are warranted to further investigate this issue.

## 5 Conclusion

The prevalence of malnutrition was high among older patients hospitalized for CAP. The independent risk factors were old age (> 74 years), past cerebral stroke, Parkinson’s disease, low serum albumin levels (< 40.5 g/L), and low hemoglobin levels (< 105 g/L). Malnutrition is associated with a worse prognosis, including prolonged hospital stay, higher in-hospital mortality, and increased readmission rates.

## Data Availability

The raw data supporting the conclusions of this article will be made available by the authors, without undue reservation.
